# Operationalizing compensation over time in neurodegenerative disease

**DOI:** 10.1093/brain/awx022

**Published:** 2017-02-23

**Authors:** Sarah Gregory, Jeffrey D. Long, Stefan Klöppel, Adeel Razi, Elisa Scheller, Lora Minkova, Marina Papoutsi, James A. Mills, Alexandra Durr, Blair R. Leavitt, Raymund A. C. Roos, Julie C. Stout, Rachael I. Scahill, Douglas R. Langbehn, Sarah J. Tabrizi, Geraint Rees

**Affiliations:** 1 Wellcome Trust Centre for Neuroimaging, Institute of Neurology, University College London, London, UK; 2 Department of Psychiatry, Carver College of Medicine, University of Iowa, Iowa, City, IA, USA; 3 Department of Biostatistics, College of Public Health, University of Iowa, Iowa City, IA, USA; 4 Albert-Ludwigs-University Freiburg, University Medical Center, Division Freiburg Brain Imaging, Freiburg, Germany; 5 Albert-Ludwigs-University Freiburg, University Medical Center, Department of Psychiatry and Psychotherapy, Freiburg, Germany; 6 University Hospital for Old Age Psychiatry, Murtenstrasse 21, 3010 Bern, Switzerland; 7 Department of Electronic Engineering, N.E.D University of Engineering and Technology, Karachi, Pakistan; 8 Albert-Ludwigs-University Freiburg, Department of Psychology, Laboratory for Biological and Personality Psychology, Freiburg, Germany; 9 Department of Neurodegenerative Disease, UCL Institute of Neurology, University College London, London, UK; 10 APHP Department of Genetics, Groupe Hospitalier Pitié-Salpêtrière, and Institut du Cerveau et de la Moelle, INSERM U1127, CNRS UMR7225, UPMC Université Paris VI UMR_S1127, Paris France; 11 Centre for Molecular Medicine and Therapeutics, Department of Medical Genetics, University of British Columbia, Canada; 12 Leiden University Medical Center, Department of Neurology, Leiden, The Netherlands; 13 School of Psychological Sciences and Institute of Clinical and Cognitive Neuroscience, Monash University, Melbourne, Australia; 14 Institute of Cognitive Neuroscience, University College London, London, UK

**Keywords:** compensation, neurodegeneration, MRI, longitudinal analysis, Huntington’s disease

## Abstract

In pre-clinical Huntington's disease, normal behaviour is maintained despite neurodegeneration, suggesting a mechanism of compensation. Gregory, Long *et al*. present two mathematical models of compensation over time and their operationalisation for neuroimaging.

## Introduction

Neuronal compensation is widely assumed to account for the dissociation between brain pathology and (absence of) behavioural change during the prodromal and early stages of neurodegenerative conditions such as Huntington’s disease and Alzheimer’s disease (Barulli and Stern, 2013; Dennis and Cabeza, 2013; Scheller *et al.*, 2014). Despite varying degrees of structural loss, patients demonstrate a level of performance during many tasks that is indistinguishable from their earlier performance, and is often similar to that of a normal population (Obeso *et al.*, 2004; Malejko *et al.*, 2014; Papoutsi *et al.*, 2014; Kloppel and Gregory, 2015). Performance is maintained until pathological factors progress and performance levels begin to deteriorate. However, neuronal mechanisms that underlie such postulated compensation in neurodegeneration are poorly understood due to the complexity in defining what compensation actually is and how it can be measured.

The characterization of compensation in neurodegeneration that we present here is derived from theoretical models of compensation in healthy ageing and Alzheimer’s disease (Lövdén *et al.*, 2010; Barulli and Stern, 2013; Reuter-Lorenz and Park, 2014). The complementary processes that may account for improved performance in the presence of structural degeneration include utilization of brain reserve and/or cognitive reserve, brain maintenance, and compensation (Barulli and Stern, 2013). Brain reserve describes the differences in brain size and structure that may support maintenance of function during ageing (or pathology). Cognitive reserve conversely is the preservation of functional efficiency and capacity despite neuronal degeneration until a critical point is reached. It is associated with lifestyle factors, including education and socio-economic status, which modulate the cognitive effects of ageing (Stern, 2006; Barulli and Stern, 2013). It is suggested that cognitive reserve comprises neuronal reserve, which accounts for the increased efficiency; and neural compensation where task-unrelated regions are recruited to perform a function (Stern, 2006). This is consistent with the concept of flexibility that, as a proxy for functional capacity and intelligence, describes the brain’s ability to optimize performance to cope with existing demands; these changes eventually leading to more permanent changes in the brain (Lövdén *et al.*, 2010).

Compensation may also represent processes where activation within existing network regions increases. This is compatible with brain maintenance, whereby susceptibility to ageing (or pathology) can impact onset of cognitive decline, and other models of compensation, which promote the concept of augmented activation in existing networks (Barulli and Stern, 2013). The Scaffolding Theory of Aging and Cognition (STAC) in particular, proposes that both brain structure and function deteriorate with age, but that compensatory scaffolding counteracts adverse effects of neuronal and functional decline (Reuter-Lorenz and Park, 2014). This is congruent with changes that occur in neurodegenerative disease where structure degenerates, but performance is maintained due to compensatory changes in brain activity. Furthermore, STAC suggests that once deterioration becomes suitably severe, compensatory effects dissipate; just as functional compensation declines as neurodegenerative pathology progresses and structural degeneration becomes too severe.

In characterizing compensation, we suppose that in a subset of prodromal patients with pathological loss of brain tissue there is reorganization within the brain that enables them to function at the same level as those without disease-related neuronal loss. As mentioned above, compensation may present as increased activation in a task-relevant brain region or recruitment of a brain region not typically associated with the function or network being tested. The latter is difficult to assess as there may be reasons for increased activity other than compensation. Furthermore, compensation may simply represent a situation whereby the rate of disease-related neuronal dysfunction is slowed over time, supporting the idea of preserved cognitive function. Here, we will focus on the notion that evidence of compensation in neurodegenerative disease is present when behaviour in patients is more similar to that of the normal population due to changes in brain activity and in the presence of structural degeneration (Barulli and Stern, 2013; Scheller *et al.*, 2014).

If compensation is defined as a lack of change in behaviour despite progressive brain pathology, then it is the absence or decreased severity of a behavioural deficit that needs to be measured as an outcome; this is challenging. In standard experimental paradigms, task-related changes in behaviour are used to explain changes in brain activity. Behavioural changes can be accounted for by concomitant changes in brain activity that ultimately differentiate the group(s) under investigation. When ‘absence’ of behavioural changes is the outcome variable, interpretation of alterations in brain activity is difficult (and sometimes impossible); we can only surmise that these changes may facilitate maintenance of normal performance. Furthermore, when investigating populations with neurodegenerative disease one might postulate an additional indeterminate effect of disease pathology on brain activity. Disease pathology may not only directly affect brain activity in terms of compensation, but may also exert subtle effects unrelated to maintenance of behaviour. Thus, it is important when attempting to operationalize compensation to try and account for pathological burden and be aware of its potential impact on the measurement of variables.

A recent review identified three components necessary to characterize compensation in ageing: extent of pathology, behavioural performance, and a measure of brain activity, such as signals derived from functional MRI measurements (Dennis and Cabeza, 2013). ‘Successful compensation’ was identified as a positive relationship between task performance and functional MRI signals, modified by age-related neuronal alterations. This model could be extended to characterize compensation in neurodegeneration. However, it does not directly account for concomitant changes in pathology across individuals during the course of neurodegenerative disease. To quantify compensatory behaviour effectively in neurodegeneration, not only should the functional MRI signal as a marker of brain activation and network-relevant task performance be explored, but it should be examined across a spectrum of pathology. We hypothesize that compensation occurs in cases where increased brain activation is needed to maintain normal levels of behaviour in the presence of structural loss. Eventually pathology becomes too severe resulting in behaviour as well as brain activation decreasing with structure over time.

**Figure 1:**
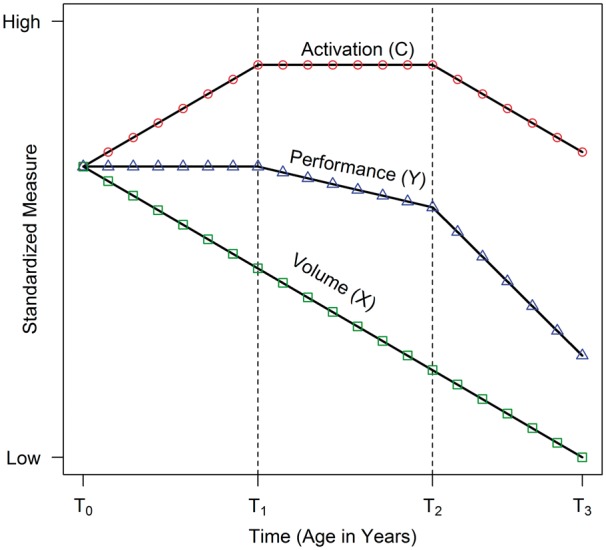
**Underlying compensation model showing change in key variables over time (activation, performance, brain volume).** Measures are assumed to be standardized to have the same mean value at the first time point. Three phases are defined by the thresholds at T_1_ and T_2_ (dashed vertical lines). Phase 1 spans (T_0_, T_1_), Phase 2 spans (T_1_ to T_2_), and Phase 3 spans (T_2_ to T_3_). Phase 1 illustrates compensation in which brain volume decreases, activation increases, and performance is maintained. Phase 2 indicates that disease effects are beginning to overwhelm compensation, as activation flattens and performance begins to decrease. Phase 3 shows the complete swamping of compensation by disease effects with all three variables decreasing.

An illustration of our hypothesized underlying model is shown in Fig. 1. The crucial components of compensation are a performance outcome (*Y*), an activation signal compensator (*C*) (e.g. functional MRI signal), and brain volume (*X*) (as a proxy for disease load). The horizontal axis represents time (or age of the participants), and the vertical axis represents scores on measures standardized to have equal means at the first observation. Curves indicate change over time for brain activity, performance, and brain volume. Three phases are depicted by the vertical dashed lines: Phase 1 spans (*T*_0_,*T*_1_), Phase 2 spans (*T*_1_,*T*_2_) and Phase 3 spans (*T*_2_,*T*_3_). In neurodegenerative disease, disease load is expected to steadily increase over time regardless of phase. Phase 1 is compensation, as brain activation (*C*) increases in reaction to brain deterioration (*X*), and performance (*Y*) is maintained. In Phase 2, disease effects start to overwhelm compensation, activation plateaus, and performance starts to deteriorate. Phase 3 shows relentless disease effects, brain activation decreases and there is acceleration in the deterioration rate of performance. The curves are idealized; there might be several stages where phasic change is monotonic rather than linear, and turns at the thresholds may be gradual rather than sharp. The important point to appreciate from Fig. 1 is that compensation leads to specific long-term patterns of change over time for three key variables.

We recently examined compensation in the TrackOn- HD cohort using a novel cross-sectional model of compensation incorporating Huntington’s disease severity, functional MRI brain activity and task performance data (Kloppel *et al.*, 2015). Results showed an asymmetric pattern of compensation within the cognitive network with evidence of a compensatory effect located in the right hemisphere, but little evidence of any compensation in the left hemisphere or in the motor network.

For cross-sectional studies, there is a degree of uncertainty regarding existing individual levels of performance, brain activity and structural load, and these findings can only suggest evidence of compensation (Raz and Lindenberger, 2011). To understand compensation in Huntington’s disease (or other neurodegenerative diseases) more fully, it is necessary to follow individuals over time. Little is understood regarding how compensatory mechanisms change over time and how they should be measured. Longitudinal models may help us to identify at what point along the disease trajectory compensatory behaviours change and eventually fail. We hypothesize that longitudinal compensation occurs when increases in brain activation over time are needed to maintain normal levels of behaviour as neuronal loss progresses. The longitudinal approach will be an extension of the cross-sectional model, so we begin with a consideration of compensation in the single time point scenario.

### Visualizing cross-sectional compensation

Visualization is a powerful tool for the analysis and interpretation of compensation patterns. Consider the case of a cross-sectional study where all the data are collected at a single time point. In terms of the underlying model (Fig. 1), the time dimension is eliminated and disease effects are inferred from differences in brain volumes among individuals. Patterns caused by compensation must be assessed using individuals sampled from the time or age spectrum. To provide examples throughout, we simulated longitudinal population data (*n* = 10 000) based on the model of Fig. 1. A time series with 21 regular visits was generated for each individual with a random intercept term to account for dependency due to repeated measures, and a random error term to account for chance perturbations; for model details see Supplementary material. To simulate the sampling of cross-sectional data, we randomly selected *n* = 200 hypothetical participants from the population, and randomly chose one time point for each individual.

**Figure 2:**
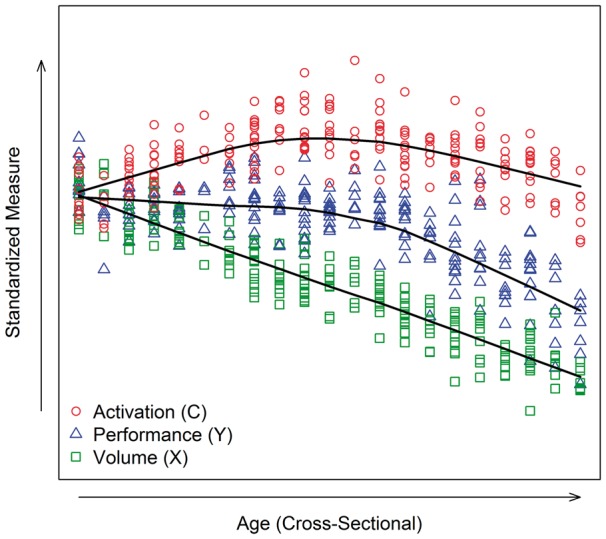
**Visualization of simulated cross-sectional data (*n* = 200) with three key variables (activation, volume, performance).** Scatterplot of values by age, with age being measured at only one time point per person. The measures were standardized to have the same mean at the first age. The smooth lines are based on a local polynomial smoother applied separately for each measure.

Figure 2 shows the sampled points using a scatterplot in which the variables are plotted as a function of age, with age being a cross-sectional variable here because there is only one age per hypothetical participant. Smooth curves (local scatterplot polynomial smoothing) were fit for each variable in isolation. The patterns of the smooth curves are reminiscent of the curves of the underlying model (i.e. Fig. 1).

### Modelling cross-sectional compensation

In addition to visualization, statistical models might be used to assess the consistency of sample data with the underlying compensation patterns and phases of Fig. 1. The underlying model specifies that the performance trajectory over age (time) is determined by disease load (brain volume) and the compensator variables (activation). One approach for assessing the agreement of cross-sectional sample data to the underlying theoretical patterns is to fit separate regression models for each of the three variables. Because the boundaries of the phases are generally unknown, our approach is to model non-linearity with a quadratic polynomial of age. Suppose that $Y_{i}$ is the performance score for the $i^{th}$ participant (i=1,…,N), and similarly for $X_{i}$ and $C_{i}$. Then consistency with the Fig. 1 patterns can be assessed by estimating the following models,

(1) $$X_{i}=\alpha_{0}+\alpha_{1}age_{i}+e_{Xi},$$

(2) $$C_{i}=\beta_{0}+\beta_{1}age_{i}+\beta_{2}age_{i}^{2}+e_{Ci},$$

and

(3) $$Y_{i}=\gamma_{0}+\gamma_{1}age_{i}+\gamma_{2}age_{i}^{2}+e_{Yi},$$

where $e_{i}$ is random error and $age_{i}$ is age measured at one time point for each person. Nuisance variables are omitted here for clarity, but they may be added to each equation for additional adjustment. For example, in Huntington’s disease, which is caused by an expansion of the cytosine-adenine-guanine (CAG) trinucleotide repeat, it is important to adjust for the length of the CAG-repeat expansion because of its well-known inverse relationship to age at motor onset (Ross and Tabrizi, 2011).

Suppose that there is adequate age representation to detect long-term patterns. Then the following parameter values are consistent with compensation patterns: $\alpha_{1}<0$ (volume constantly decreasing), $\beta_{2}<0$ (activation having a concave-downward pattern), and $\gamma_{2}<0$ (performance having a concave-downward pattern). The Equation 1–3 parameters can be estimated with multiple regression using ordinary least-squares and inference is predicated on the assumptions of normally distributed and homogeneous error. A one-sided *t*-test can be used to evaluate the null hypothesis that each parameter is equal to zero, with the alternative hypothesis that a parameter is less than zero. These tests, along with visualization, are the primary means of assessing consistency of cross-sectional data with long-term compensation effects.

Additional inferences are possible if one is willing to assume that Equations 1 and 2 are true models, rather than just approximations for the Fig. 1 patterns. We hypothesize that Y is determined by X and C allowing for random error. It follows that Equation 3 is a linear combination of the first two equations $Y_{i}=X_{i}+C_{i}+e_{Yi}$. The equivalence implies $\gamma_{0}=a_{0}+\beta_{0}$, $\gamma_{1}=\alpha_{1}+\beta_{1}$, and $\gamma_{2}=\beta_{2}$. The latter two equivalencies are most important for compensation, and a confidence interval for the difference of parameters can be computed based on the sample estimates; that is, a confidence interval for $\gamma_{1}-(\alpha_{1}+\beta_{1})$ and a confidence interval for $\gamma_{2}-\beta_{2}$. Evidence for compensation patterns is provided when 0 is contained in each confidence interval, indicating the sample difference is not statistically reliable. A more lax criterion for consistency with the compensation of Fig. 1 is that the second confidence interval does not contain negative values. Figure 1 implies that the quadratic coefficient should be stronger for C (greater downward concavity), so that $-\beta_{2}>\gamma_{2}$. Similarly, $\gamma_{1}$ is the sum of a positive value ($\beta_{1}$) and a negative value ($\alpha_{1}$) so that $\beta_{1}>\gamma_{1}$, which can also be evaluated with a confidence interval.

A method for simultaneously estimating all parameters with ordinary least-squares and using standard errors based on the covariance of the parameters is provided in the Supplementary material. More sophisticated approaches are possible, such as estimating the phase thresholds based on visualization, for example, and then using piece-wise or spline models of age for the C and Y regression models (X has a constant decrease and does not need splines).

### Visualizing longitudinal compensation

Using cross-sectional data to make inferences about a longitudinal process is not optimal. Valid inferences depend on the extent to which individuals of different ages accurately represent the general process that all people experience over time. This exchangeability is not plausible when there are cohort effects, such as when a new treatment is only available to young patients. Furthermore, between-individual variability tends to be larger than within-individual variability, often resulting in higher statistical power for testing effects when participants are measured over time. For these reasons, longitudinal data are preferred for examining and testing compensation patterns.

**Figure 3:**
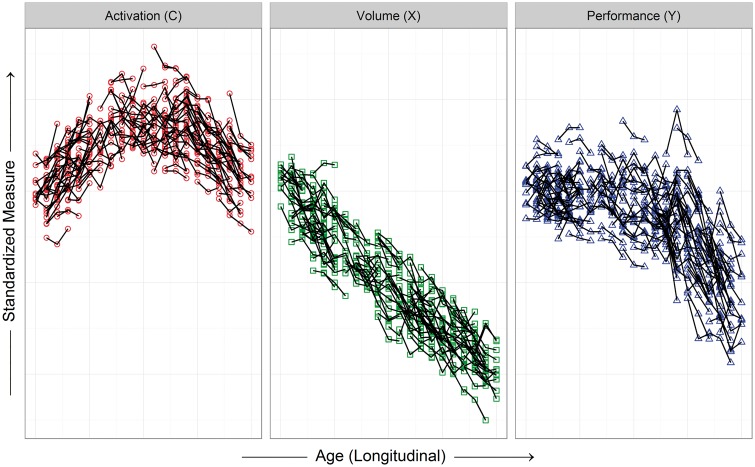
**Visualization of simulated longitudinal sample data (*n* = 200, three time points) with three key variables (activation, volume, performance).** The spaghetti plots connect the three repeated measures for each individual with a line; each variable is depicted in a different panel (same participants in each), and all the variables are standardized to have the same mean for the first age.

Longitudinal sample data are assumed to arise when individuals are randomly sampled from a population (here with neurodegenerative disease) and their responses are recorded over time. This process was simulated by randomly sampling *n* = 200 individuals from our generated population and randomly selecting three consecutive time points from the 21 available. Assuming the symbols in Fig. 1 represent observations at annual visits, the simulated sample data represent a 3-year observational study in which participants vary extensively on their age at entry. Figure 2 can be used for visualization, but this ignores the serial nature of the data. More appropriately, a spaghetti plot can be constructed in which the repeated measures of each participant are connected by lines. Figure 3 shows the spaghetti plot for the simulated sample data, with the measures panelled to facilitate interpretation. Variability among individual trajectories is apparent due to the random effect and the random error, but the patterns are similar to Fig. 1. A smooth curve can be fitted among the individual trajectories to characterize aggregate change, either with a scatterplot smoother or with the methods discussed below.

### Modelling longitudinal compensation

When repeated measurements are available, it is natural to extend the model of Equations 1–3 to a longitudinal context. The approach here is to adopt the same model form as Equations 1–3, but account for the correlation induced by repeated measurements using random effects and an error term for random perturbations. The general framework is the linear mixed model (LMM).

Suppose that $Y_{ij}$ is the performance outcome for the $i^{th}$ participant at the $j^{th}$ age ($j=1,\ldots ,T_{i})$, with $T_{i}=T$ when there are no missing data. Similarly for $X_{ij}$ and $C_{ij}$, the LMM version of the compensation pattern models are

(4) $$X_{ij}=a_{0i}+\alpha_{0}+\alpha_{1}age_{ij}+e_{Xij},$$

(5) $$C_{ij}=b_{0i}+\beta_{0}+\beta_{1}age_{ij}+\beta_{2}age_{ij}^{2}+e_{Cij},$$

and

(6) $$Y_{ij}=g_{0i}+\gamma_{0}+\gamma_{1}age_{ij}+\gamma_{2}age_{ij}^{2}+e_{Yij}.$$

Nuisance variables are again suppressed for clarity. In Equations 4–6, age is now time-varying; the Greek letters are fixed effects that do not vary over time or participants; the lower case Arabic letters (other than *e*) are individual-specific random effects that vary over participants, but not time (assumed to be normally distributed with zero-mean and non-zero variance); and $e_{ij}$ is random error (assumed to be normally distributed with zero-mean and constant variance over time). The variance-covariance matrix among the times (ages) for the outcome variable is a function of the variance components of the random effects and error. The single random effect results in a constant covariance between any two time points, but additional random effects can be specified to provide a richer structure (Verbeke, 2000).

Similar to the cross-sectional context, long-term patterns consistent with compensation would have $\alpha_{1}<0$ (volume constantly decreasing), $\beta_{2}<0$ (activation having a concave-downward pattern over time), and $\gamma_{2}<0$ (performance having a concave-downward pattern over time).

A method for simultaneously estimating the parameters of Equations 2–4 is provided in the Supplementary material. Maximum likelihood methods are used with LMM, allowing similar tests of estimated coefficients and confidence intervals as in the cross-sectional case.

## Discussion

The longitudinal compensation models proposed here are first attempts at operationalizing compensation over time. We argue that to analyse compensatory mechanisms, it is necessary to model changes in brain activity and disease load (pathological severity), which are thought to influence changes in performance. Incorporating structural measures of disease load within the compensation model allows an index of disease progression and an account of variability in structural degeneration. Combining disease load with brain activation and performance constitutes novel longitudinal compensation models that provide a means for empirical testing of longitudinal compensation in neurodegeneration.

There are some considerations and potential limitations to longitudinal compensation modelling. First, our examples of simulated sample data are idealized; we randomly sampled individuals from the entire spectrum of the critical age epoch to illustrate compensation patterns. Restricting the age (or disease load) range has implications for the statistical compensation models. For example, if participants are only sampled from Phase 1 of our underlying model (Fig. 1), the regression coefficients might be severely attenuated relative to sampling from the entire range. Under the Phase 1 sampling scenario, non-linear effects probably cannot be detected and performance does not vary over time and cannot covary with age (or activation or disease load). If only Phase 3 participants are sampled, the linear age effects might be very strong, but again the non-linear effects indicative of long-term compensation probably cannot be detected. In planning a study or analysing data from an existing database, it is important to assess the extent of sampling over the critical epoch in which compensation patterns are expected to emerge. For example, in Huntington’s disease research, the critical epoch is from the pre-manifest stage (prior to motor diagnosis) to early Huntington’s disease (up to a few years post motor diagnosis). As Huntington’s disease is a relatively slow progressing disease, it is important to sample both pre-manifest and early Huntington’s disease participants to increase the likelihood of detecting patterns of the hypothesized underlying model. Similar considerations apply for other diseases.

Variable transformations must be carefully considered when studying compensation. Transformations are routine in many research areas; examples include scaling a brain substructure volume (e.g. putamen volume) by intracranial volume (ICV), and scaling a performance measure based on an underlying item response theory model (e.g. the Rasch model). Our compensation model assumes particular linear and curvilinear functional forms for variables over time. Non-linear transformations of variables applied at each time point can induce inconsistency with the patterns of Fig. 1, even though the untransformed trajectories have the exact patterns over time. For example, a constant decrease in putamen volume is consistent with our underlying model. Putamen volume divided by baseline ICV is expected to have a constant decrease over time because division (multiplication) is a linear transformation. On the other hand, non-linear corrections for ICV have been suggested, such as putamen volume divided by baseline ICV taken to the $b^{th}$ power (i.e. $ICV^{b}$, where b∉{0,1}) (Liu *et al.*, 2014). Such transformations may induce a curvilinear decrease over time, which is inconsistent with our underlying model (there may be monotonic decrease but not linear decrease). It is possible to sketch expected compensation trajectories of transformed variables over time. However, the graphical and statistical methods for examining the extent of compensation might vary from the ones discussed here.

Although we think the examination of compensation can be performed with the graphical methods discussed, it is acknowledged that statistical testing is widespread. In the statistical evaluation of compensation, sample size and the number of repeated measures should be considered when testing effects and computing confidence intervals. Sample size can have potentially opposite effects on the assessment of compensation patterns, depending on the method that is used. If linear and quadratic coefficients are tested for significance in either Equations 1–3 or 4–6, then a larger sample size will increase statistical power and increase the likelihood of rejecting null hypotheses of non-compensation (all other things being equal). On the other hand, if confidence intervals of differences of parameters are computed, then a large sample size will cause smaller intervals (other things being equal) and potentially small clinical differences can become significant statistical differences. To address this issue, it would be helpful to have a threshold based on an important clinical difference. However, defining such a threshold is challenging. Another consideration for our simulation is that the error variance and random effects variance were deliberately small in order to illustrate compensation patterns. Real sample data may be nosier and may not have the orderliness depicted in our graphs.

While there is evidence that neuronal degeneration becomes increasingly widespread anatomically as disease progresses, there may be a high level of individual variability in the rate of change of degeneration. Although this may be a negligible consideration for observation periods that are short relative to disease evolution, it might considerably impact the change in compensatory processes between individuals, such that one individual may deteriorate significantly faster than another with comparable baseline disease load and functional MRI activity. Our longitudinal statistical model (Equations 4–6) accounts for individual variability of initial levels (i.e. random intercepts), but it may be necessary to add random effects for linear and quadratic terms in order to adequately account for the variation. It should be noted that adding random effects will increase estimation complexity, and large sample sizes may be necessary for proper inferences.

The simulated longitudinal data (Fig. 3) depict the situation in which many individuals are tracked for a relatively short time. To increase the likelihood of avoiding potential cohort effects and to better understand longitudinal evolution, it is desirable to sample fewer individuals, but follow them for a long time (e.g. 10 years). Typical study resources do not allow for follow-up of more than a few years, so sampling approaches should be devised to ensure adequate between-individual differences.

It is also important to note that while we have focused here on brain volume as a measure of disease load in neurodegeneration, the compensation models we present can easily be adapted to include alternative measures of disease load or ones that are most appropriate for the disorder being examined. For example, while structural volume is the most robust measure for Huntington’s disease progression, the boundary shift integral, a measure of cerebral volume change, could also be used as an alternative measure of disease load (Freeborough and Fox, 1997). This also applies to measures of brain activity where electrophysiological measures or other MRI measures could replace functional MRI signals.

## Conclusion

Compensation is proposed to account for the dissociation between progression of neuronal pathology and absence of behavioural changes in the early stages of neurodegeneration. Here, we have provided a framework for the operationalization of compensation. The focus of analysis—both statistical modelling and visualization—is on patterns of change caused by compensation over a critical epoch. Although these are not the only possible models of longitudinal compensation, we propose that the field should adopt a more systematic approach to operationalizing and investigating compensation using similar theoretical and operational approaches to those included here.

## Supplementary Material

Supplementary Data
