# Supplementary material for: Operationalizing compensation over time in neurodegenerative disease
Source: Brain. 2017 Feb 23;140(4):1158–65. doi: 10.1093/brain/awx022 (PMC5382953; doi:10.1093/brain/awx022)
Supplement: Supplementary Data [file awx022_supp.pdf]

# Supplementary Information

## Simulated Population Data

Population data were simulated according to Figure 1 of the main text. Figure 1 depicts the situation in which the Y, C, and X variables have different relationships for three phases. A regression spline approach was used for the simulation in which Y was a function of C and X, with regression coefficients varying by phase and error being allowed for the predictor variables. Suppose that $Y_{ij}$ is the performance outcome for the $i^{th}$subject ($i = 1, \ldots, N$) at the $j^{th}$ time point ($j = 1, \ldots, T_i$), with $T_i = T$, so there was no missing data for the simulation.

The underlying model expresses Y as a function of C and X, so the latter two were first generated consistent with their pattern in Figure 1 allowing for errors in the variables. A linear mixed model (LMM) was used with a random intercept term and random error. X is a linear function of time, and the LMM for the simulation was

$$X_{ij} = \alpha_0 + a_{0i} + \alpha_1 t_{ij} + e_{ij}.$$

We assume $a_{0i} \sim \mathcal{N}(0, \sigma^2_{a_{0i}})$ and $e_{ij} \sim \mathcal{N}(0, \sigma^2_e)$. The variables were standardized to have the same initial means, so we set $\alpha_0 = 0$. The other parameters were set to $\alpha_1 = -1$, $\sigma^2_{a_{0i}} = 0.05$, and $\sigma^2_e = 0.01$. Time values ranged from 0 to 2 incremented by a tenth, but the values were not intended to represent a specific metric (no values or units were used in the main text). A random number generator was used to obtain realized values of the random effect for each hypothetical subject, and the random error for each subject at each time point.

Figure 1 indicates that C has a nonlinear trend. Therefore, C was a spline function of time,

$$C_{ij} = \psi_0 + p_{0i} + \psi_1 \left(t_{ij} - T_1\right)_{t_{ij} \geq T_1} + \psi_2 \left(t_{ij} - T_2\right)_{t_{ij} \geq T_2} + e_{ij}.$$

The parameters were set to $\psi_0 = 0, \psi_1 = \psi_2 = -1, \sigma^2_{p_{0i}} = 0.05$, and $\sigma^2_e = 0.01$.

For the simulation of Y, let us define

$$C^{(1)}_{ij} = \begin{cases} C_{ij} if\ t_{ij} \in [T_0, T_1) \\ 0\ otherwise \end{cases}, C^{(2)}_{ij} = \begin{cases} C_{ij} if\ t_{ij} \in [T_1, T_2) \\ 0\ otherwise \end{cases}, C^{(3)}_{ij} = \begin{cases} C_{ij} if\ t_{ij} \in [T_2, T_3] \\ 0\ otherwise \end{cases},$$

and likewise for $X^{(1)}_{ij}, X^{(2)}_{ij}, X^{(3)}_{ij}$. Then the expected value equations for the phases are

$$E\left(Y_{ij} \middle| t_{ij} \in [T_0, T_1)\right) = \gamma_0 + \gamma_1 C^{(1)}_{ij} + \gamma_2 X^{(1)}_{ij},$$

$$E\left(Y_{ij} \middle| t_{ij} \in [T_1, T_2)\right) = \delta_0 + \delta_1 C^{(2)}_{ij} + \delta_2 X^{(2)}_{ij},$$

$$E\left(Y_{ij} \middle| t_{ij} \in [T_1, T_2]\right) = \pi_0 + \pi_1 C^{(3)}_{ij} + \pi_2 X^{(3)}_{ij}.$$

Because Y is flat over Phase 1, we set $\gamma_0 = \gamma_1 = \gamma_2 = 0$. Constraining the curves to have a smooth transition at the phase thresholds resulted in the expected value formula

$$EY_{ij} = [\delta_1(C_{ij} - C_{T_1}) + \delta_2(X_{ij} - X_{T_1})] \cdot I(t \in [T_0, T_1)) + [\delta_1(C_{T_2} - C_{T_1}) + \delta_2(X_{T_2} - X_{T_1}) + \pi_1(C_{ij} - C_{T_2})) + \pi_2(X_{ij} - X_{T_2})] \cdot I(t \in [T_1, T_2]),$$

where $I(\cdot)$ equal 1 if the condition inside the parentheses is met, and 0 otherwise. Adding a random intercept ($g_{0i}$) and random error, the simulated value of Y was

$$Y_{ij} = EY_{ij} + g_{0i} + e_{ij}.$$

The parameter values were set to $\delta_1 = -0.1, \delta_2 = 0.4, \pi_1 = 0.9, \pi_2 = 0.8, \sigma^2_{g_{0i}} = 0.05, \sigma^2_e = 0.01$. Simulated values of X and C from the previous step were used along with values from the random number generator (for $g_{0i}, e_{ij}$), and $Y_{ij}$was computed. A population of $N = 10000$ was generated. Cross-sectional data was generated by randomly sampling 200 subject from the population and then random selecting a single time point for each person. Longitudinal data was generated by the same process except 3 sequential time points were selected for each subject. R code for simulating the data can be obtained from the authors.

## Simultaneous Parameter Estimation

The estimation and CI approaches in the main text present separate regression models for the three variables of interest $(X, C, Y)$. It is possible to estimate the parameters of all three regression models simultaneously and then test individual estimates and compute CIs for the key parameter differences with SEs that take the covariance among the parameters into account. We propose a *stacked model* approach in which the vectors of the three variables are concatenated (stacked) and dummy coding is used on the predictor side to specify the model. First consider cross-sectional data in which we have $N$ scores for each variable. The stacked model in matrix notation is

$$\mathbb{Y} = \mathbb{D}_{int}\boldsymbol{\delta} + \mathbb{D}_{linear}\boldsymbol{\eta} + \mathbb{D}_{quad}\boldsymbol{\theta} + \boldsymbol{e}$$

where $\mathbb{Y}$ is a $3N \times 1$ vector consisting of the stacked scores of $X, C, Y$; $\mathbb{D}_{int}$ is a $3N \times 3$ design matrix for the intercept terms with associated intercept parameter vector $\boldsymbol{\delta}$; $\mathbb{D}_{linear}$ is a $3N \times 3$ design matrix for the linear age terms with associated parameter vector $\boldsymbol{\eta}$; $\mathbb{D}_{quad}$is a $3N \times 3$ design matrix for the age-squared (quadratic) terms with associated parameter vector $\boldsymbol{\theta}$; and $\boldsymbol{e}$ is the $3N \times 3$ matrix of random error terms. The matrices are specified in the following manner:

$$\begin{bmatrix} X_1 \\ \vdots \\ X_N \\ C_1 \\ \vdots \\ C_N \\ Y_1 \\ \vdots \\ Y_N \end{bmatrix} = \begin{bmatrix} 1 & 0 & 0 \\ \vdots & \vdots & \vdots \\ 1 & 0 & 0 \\ 0 & 1 & 0 \\ \vdots & \vdots & \vdots \\ 0 & 1 & 0 \\ 0 & 0 & 1 \\ \vdots & \vdots & \vdots \\ 0 & 0 & 1 \end{bmatrix} \begin{bmatrix} \alpha_0 \\ \beta_0 \\ \gamma_0 \end{bmatrix} + \begin{bmatrix} age_1 & 0 & 0 \\ \vdots & \vdots & \vdots \\ age_N & 0 & 0 \\ 0 & age_1 & 0 \\ \vdots & \vdots & \vdots \\ 0 & age_N & 0 \\ 0 & 0 & age_1 \\ \vdots & \vdots & \vdots \\ 0 & 0 & age_N \end{bmatrix} \begin{bmatrix} \alpha_1 \\ \beta_1 \\ \gamma_1 \end{bmatrix} + \begin{bmatrix} 0 & 0 & 0 \\ \vdots & \vdots & \vdots \\ 0 & 0 & 0 \\ 0 & age_1^2 & 0 \\ \vdots & \vdots & \vdots \\ 0 & age_N^2 & 0 \\ 0 & 0 & age_1^2 \\ \vdots & \vdots & \vdots \\ 0 & 0 & age_N^2 \end{bmatrix} \begin{bmatrix} 0 \\ \beta_2 \\ \gamma_2 \end{bmatrix} + \begin{bmatrix} e_{Xi} \\ \vdots \\ e_{XN} \\ e_{Ci} \\ \vdots \\ e_{CN} \\ e_{Yi} \\ \vdots \\ e_{YN} \end{bmatrix}$$

The matrix equation yields the individual variable models of Equations 1-3 in the text and parameter estimate can be calculated via ordinary least squares. A test of the null hypothesis for an individual parameter, e.g. $H_0: \beta_2 = 0$, is based on the t-ratio,

$$t = \frac{\hat{\beta}_2}{SE(\hat{\beta}_2)}$$

Regarding CIs for differences of parameters, we make use of covariance among parameters in computing the SEs and CIs. The first contrast of interest is the difference of the performance ($Y$) linear term and the sum of the linear terms of the other two variables, i.e., $\gamma_1 - (\alpha_1 + \beta_1)$. The large-sample 95% CI is

$$\hat{\gamma}_1 - \hat{\alpha}_1 - \hat{\beta}_1 \pm 1.96 \cdot SE(\hat{\gamma}_1 - \hat{\alpha}_1 - \hat{\beta}_1).$$

The second difference between the quadratic term of performance and the compensatory variable, $\gamma_2 - \beta_2$. The CI is

$$\hat{\gamma}_2 - \hat{\beta}_2 \pm 1.96 \cdot SE(\hat{\gamma}_2 - \hat{\beta}_2).$$

Once the design matrices have been constructed, standard statistical can be used for OLS estimation. The caveat is that the default intercept needs to be suppressed because it is specified through $\mathbb{D}_{int}$. Suppose that the design matrices are named D.int, D.linear, and D.quad, and the stacked response vector is Y. Then the linear model is estimated in the R software using the syntax lm(Y ~ 0 + D.int + D.linear + D.quad), where 0 suppresses the default intercept.

Longitudinal data is handled in an analogous way, with extensions made for the linear mixed model (LMM). The stacked LMM is

$$\mathbb{Y} = \mathbb{D}_{int}\boldsymbol{\delta} + \mathbb{D}_{linear}\boldsymbol{\eta} + \mathbb{D}_{quad}\boldsymbol{\theta} + \mathbb{D}_{re}\boldsymbol{\zeta} + \boldsymbol{e}$$

where $\mathbb{D}_{re}$ is a design matrix for the random effects with associated vector $\boldsymbol{\zeta}$. We consider only random intercepts, but additional random effects can be specified. The LMM for longitudinal data has the important feature that $\mathbb{Y}$ is now a stacked vector of repeated measures and subjects. We now have $\boldsymbol{X}_i^T = [X_{i1}, \dots, X_{in_i}]$, and similarly for $C$ and $Y$. If there is no missing data, then $n_i = n$ and the length of the stacked $X$ vector is $nN$, and similarly for the other two variables, so that $\mathbb{Y}$ is a $3nN$ row vector. Similar adjustments are made for the other matrices so that all row dimensions are $3nN$ (again, with no missing data). The specifics of the stacked matrices are as follows. Consider the overly simple case of two subjects ($i = 1,2$) each with two repeated measures ($j = 1,2$). Then the matrix equation is the following,

$$
\begin{bmatrix} X_{11} \\ X_{12} \\ X_{21} \\ X_{22} \\ C_{11} \\ C_{12} \\ C_{21} \\ C_{22} \\ Y_{11} \\ Y_{12} \\ Y_{21} \\ Y_{22} \end{bmatrix}
=
\begin{bmatrix} 100 \\ 100 \\ 100 \\ 100 \\ 010 \\ 010 \\ 010 \\ 010 \\ 001 \\ 001 \\ 001 \\ 001 \end{bmatrix}
\begin{bmatrix} \alpha_0 \\ \beta_0 \\ \gamma_0 \end{bmatrix}
+
\begin{bmatrix}
age_{11} & 0 & 0 \\
age_{12} & 0 & 0 \\
age_{21} & 0 & 0 \\
age_{22} & 0 & 0 \\
0 & age_{11} & 0 \\
0 & age_{12} & 0 \\
0 & age_{21} & 0 \\
0 & age_{22} & 0 \\
0 & 0 & age_{11} \\
0 & 0 & age_{12} \\
0 & 0 & age_{21} \\
0 & 0 & age_{22}
\end{bmatrix}
\begin{bmatrix} \alpha_1 \\ \beta_1 \\ \gamma_1 \end{bmatrix}
+
\begin{bmatrix}
0 & 0 & 0 \\
0 & 0 & 0 \\
0 & 0 & 0 \\
0 & 0 & 0 \\
0 & age_{11}^2 & 0 \\
0 & age_{12}^2 & 0 \\
0 & age_{21}^2 & 0 \\
0 & age_{22}^2 & 0 \\
0 & 0 & age_{11}^2 \\
0 & 0 & age_{12}^2 \\
0 & 0 & age_{21}^2 \\
0 & 0 & age_{22}^2
\end{bmatrix}
\begin{bmatrix} 0 \\ \beta_2 \\ \gamma_2 \end{bmatrix}
$$

$$
+
\begin{bmatrix}
1 & 0 & 0 & 0 & 0 & 0 \\
1 & 0 & 0 & 0 & 0 & 0 \\
0 & 1 & 0 & 0 & 0 & 0 \\
0 & 1 & 0 & 0 & 0 & 0 \\
0 & 0 & 1 & 0 & 0 & 0 \\
0 & 0 & 1 & 0 & 0 & 0 \\
0 & 0 & 0 & 1 & 0 & 0 \\
0 & 0 & 0 & 1 & 0 & 0 \\
0 & 0 & 0 & 0 & 1 & 0 \\
0 & 0 & 0 & 0 & 1 & 0 \\
0 & 0 & 0 & 0 & 0 & 1 \\
0 & 0 & 0 & 0 & 0 & 1
\end{bmatrix}
\begin{bmatrix} a_{01} \\ a_{02} \\ b_{01} \\ b_{02} \\ g_{01} \\ g_{02} \end{bmatrix}
+
\begin{bmatrix} e_{11} \\ e_{12} \\ e_{21} \\ e_{22} \\ e_{11} \\ e_{12} \\ e_{21} \\ e_{22} \\ e_{11} \\ e_{12} \\ e_{21} \\ e_{22} \end{bmatrix}.
$$

The LMM parameter estimates are obtained via likelihood methods. Similar CIs as proposed for the cross-sectional model can be calculated. Standard LMM software can be used suppressing the default intercept. Suppose that the design matrices are named D.int, D.linear, D.quad, and D.rea, nd the stacked response vector is Y. Then the LMM is estimated in R with the lme4 package using the syntax lmer(Y ~ 0 + D.int + D.linear + D.quad + (1 | subject)).
